# The myxobacterial metabolite ratjadone A inhibits HIV infection by blocking the Rev/CRM1-mediated nuclear export pathway

**DOI:** 10.1186/1475-2859-13-17

**Published:** 2014-01-29

**Authors:** Eric Fleta-Soriano, Javier P Martinez, Bettina Hinkelmann, Klaus Gerth, Peter Washausen, Juana Diez, Ronald Frank, Florenz Sasse, Andreas Meyerhans

**Affiliations:** 1 Infection Biology Group, Department of Experimental and Health Sciences, Universitat Pompeu Fabra, Dr. Aiguader 88 08003, Barcelona, Spain; 2 Department of Chemical Biology, Helmholtz Centre for Infection Research, Braunschweig, Germany; 3 Molecular Virology Group, Department of Experimental and Health Sciences, Universitat Pompeu Fabra, Barcelona, Spain; 4 Department of Microbial Drugs, Helmholtz Centre for Infection Research, Braunschweig, Germany; 5 Institució Catalana de Recerca i Estudis Avançats (ICREA), Barcelona, Spain

**Keywords:** Ratjadone, Myxobacteria, HIV, Rev, Host factor, CRM1, Nuclear export

## Abstract

**Background:**

The nuclear export of unspliced and partially spliced HIV-1 mRNA is mediated by the recognition of a leucine-rich nuclear export signal (NES) in the HIV Rev protein by the host protein CRM1/Exportin1. This makes the CRM1-Rev complex an attractive target for the development of new antiviral drugs. Here we tested the anti-HIV efficacy of ratjadone A, a CRM1 inhibitor derived from myxobacteria.

**Results:**

Ratjadone A inhibits HIV infection *in vitro* in a dose-dependent manner with EC_50_ values at the nanomolar range. The inhibitory effect of ratjadone A occurs around 12 hours post-infection and is specific for the Rev/CRM1-mediated nuclear export pathway. By using a drug affinity responsive target stability (DARTS) assay we could demonstrate that ratjadone A interferes with the formation of the CRM1-Rev-NES complex by binding to CRM1 but not to Rev.

**Conclusion:**

Ratjadone A exhibits strong anti-HIV activity but low selectivity due to toxic effects. Although this limits its potential use as a therapeutic drug, further studies with derivatives of ratjadones might help to overcome these difficulties in the future.

## Introduction

**Figure 1:**
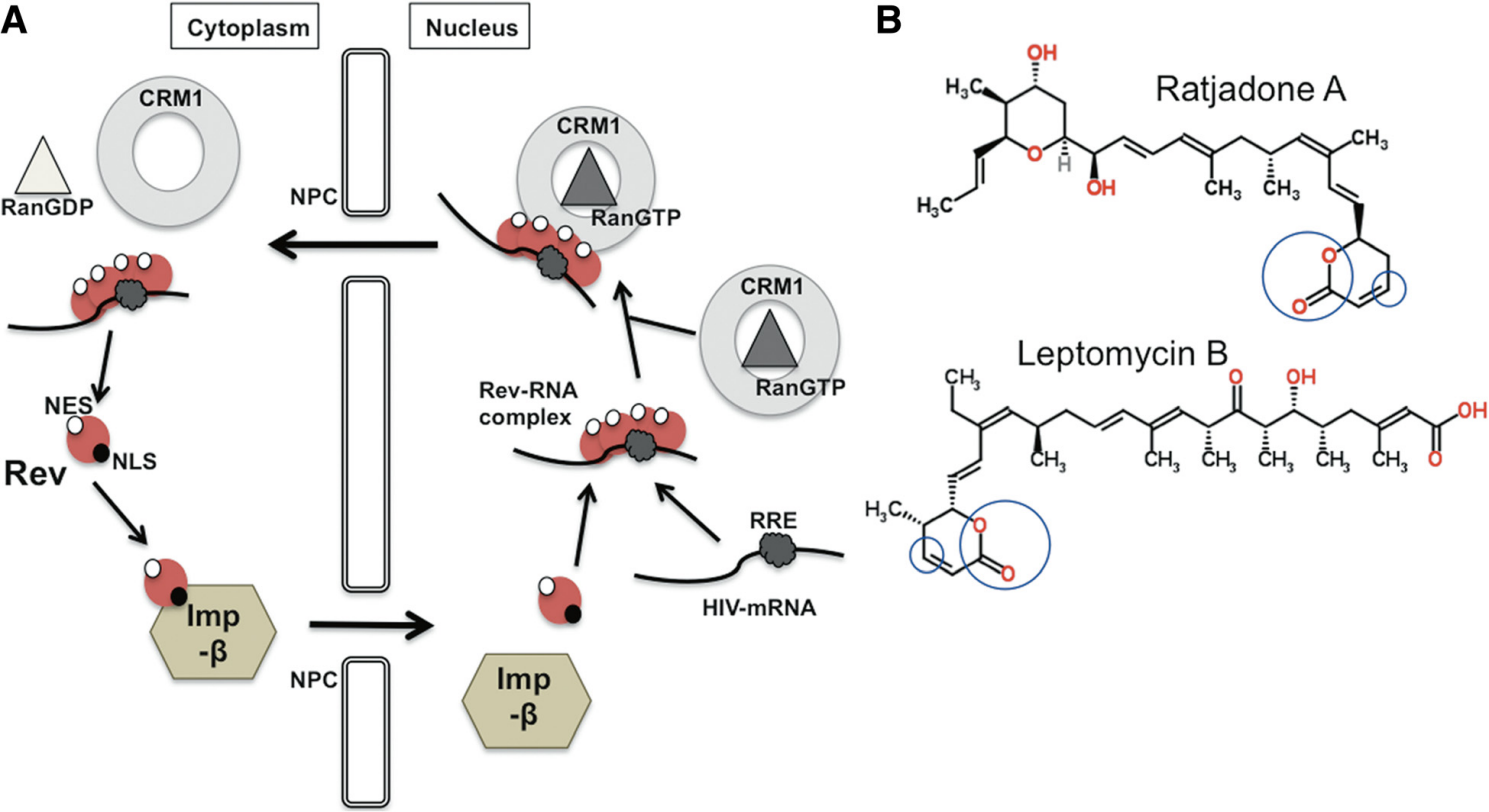
**Schematic representation of Rev-mediated nuclear export of HIV mRNAs and chemical structures of RaTA and LMB. (A)** Binding of the Nuclear Localization Signal (NLS) in Rev to importin-β (Imp-β) triggers the nuclear internalization of Rev through the Nuclear Pore Complex (NPC). Once in the nucleus, Rev binds to the Rev-responsive element (RRE) of the HIV mRNA. This interaction exposes the Nuclear Export Signal (NES) of Rev for recognition by CRM1. The CRM1-Rev-mRNA complex is stabilized by the phosphorylated form of Ran (RanGTP) and crosses the nuclear pore into the cytoplasm where Ran is dephosphorylated (RanGDP) and the complex is disassembled making HIV mRNAs available for translation. **(B)** The specific groups within the chemical structures of RaTA and LMB that are involved in the interaction with CRM1 are circled. A complete description of these interactions can be found in (55). Structures are freely available from: http://www.chemspider.com. Chemspider IDs: 5293127 and 4948244.

The HIV-encoded Rev protein is a trans-acting nuclear factor that plays a pivotal role in the virus post-transcriptional regulation [1]. Through specific interactions with RanGTPase-dependent cellular mediators, Rev mediates the transport of unspliced and partially spliced viral mRNAs to the host-cell cytoplasm (Figure 1A). Rev shuttles in and out of the nucleus via two distinct signals: an arginine-rich nuclear localization signal (NLS) in the Rev RNA-binding domain, and a leucine-rich nuclear export signal (NES) found in the Rev-activation domain [2]. Rev enters the nucleus by direct binding of its NLS to the human nuclear import receptor, importin-β [3]. Inside the nucleus, Rev binds to the Rev-responsive element (RRE)-containing HIV mRNAs forming a complex that hides the Rev NLS signal and exposes its NES for recognition by the nuclear export mediator Exportin1/CRM1 [4]. This interaction triggers the transfer of unspliced and partially spliced viral mRNAs to the cytoplasm [5,6] for the subsequent translation of HIV structural proteins and encapsidation.

A functional Rev-CRM1 cooperation is critical for HIV replication [3,7-12]. Although several nuclear export mediators have been described [4], the CRM1 pathway is selectively used for the Rev-dependent nuclear export of HIV-1 mRNAs [2]. Deletions in both the N- and C-terminal domains of Rev impair virus replication [13] and Rev mutants that are unable to multimerize do not localize to the nucleus [14,15]. Similarly, both importin-β and CRM1 mutants have been shown to block nuclear import and export pathways despite a functional NES-carrying partner [16-19]. Treatment with CRM1 inhibitors, such as leptomycin B (LMB) [19,20], has shown to block HIV replication *in vitro*[7,8], although toxicity-related effects has hampered further development of these compounds as antiviral drugs.

In this work we report the anti-HIV efficacy of ratjadone A (denoted here as RaTA), a CRM1 inhibitor produced by the myxobacterium *Sorangium cellulosum*[21] that shares structural similarities with LMB (Figure 1B). Myxobacteria are a group of bacteria from soil that can be considered as microbial cell factories. Their fermentation has been optimized to improve the yield and diversity of large amounts of secondary metabolites with interesting biological functions [22-24]. The anti-HIV activity of these metabolites has recently been evaluated by a high-throughput two-step infectivity assay [25]. Amongst the hits were ratjadones A, B, C and D that previously have been shown to exhibit antibiotic effects against fungi and growth inhibition of mammalian cancer cells with the same mode of action as LMB [26]. However, RaTA has been suggested to be more potent and less toxic than LMB [27]. These differences in activities between RaTA and LMB could be due to structural changes at carbon C8 and a tale of different size and polarity (Figure 1B) [27]. Thus, putative anti-HIV effects of RaTA may differ from LMB. We show here that RaTA inhibits HIV replication *in vitro* in a dose-dependent manner with higher potency than LMB. RaTA interferes with the HIV mRNA nuclear export step by binding to CRM1 but not to Rev.

## Results

### Ratjadone A (RaTA) inhibits HIV infection *in vitro*

**Figure 2:**
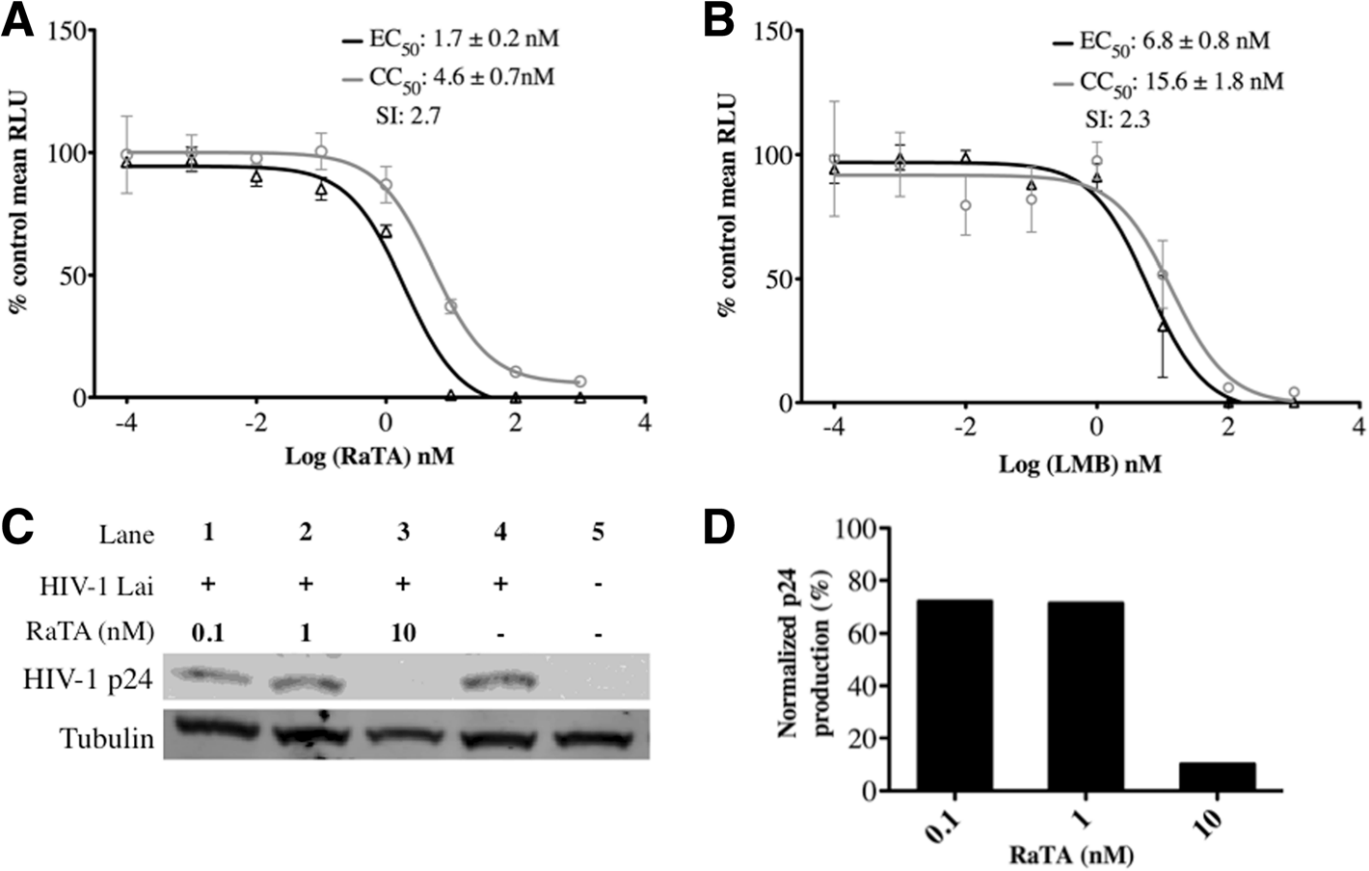
**Potency of ratjadone A against HIV infection of TZM-bl cells.** Cells were seeded in 96-well plates in triplicate and treated with increasing concentrations of RaTA **(A)** or LMB **(B)** and infected with HIV_LAI_ at a multiplicity of infection (MOI) of 0.5. 48 h after infection cells were assayed for luciferase activity and the mean relative light units (RLU) were plotted as % relative to DMSO (vehicle) for both infectivity and cell viability. Effective Concentration 50 (EC_50_) and Cytotoxic Concentration 50 (CC_50_) were estimated by non-linear regression of log inhibitor vs. normalized response and used to calculate the Selectivity Index (SI) value (see Materials and methods). Bars: standard error of the mean (SEM). For panels **(C)** and **(D)**, cells were HIV infected by spinoculation, seeded in 6-well plates and treated with increasing concentrations of RaTA. 48 h after infection, cells were lysed and analysed by Western Blotting **(C)**. HIV p24 bands are shown in the upper row. Lanes 1 to 3: cells incubated with RaTA at different concentrations; Lane 4: DMSO control; Lane 5: uninfected DMSO control. Tubulin was used as a loading control (lower row). Every band in panel C was normalized respect to the loading control and quantified. The relative p24 production was plotted as % relative to DMSO control **(D)**. The drug solvent concentration (0.1% DMSO) in every sample was constant.

To analyse the antiviral potency of RaTA, a TZM-bl infectivity test was performed as previously described [25]. The TZM-bl cell-based assay measures the antiviral activity of small molecules as a function of reductions in the HIV Tat-regulated luciferase reporter gene expression [28]. Cells were pre-treated with 10-fold serial dilutions of RaTA or LMB as control and infected with HIV_LAI_ at a MOI of 0.5. HIV-dependent luciferase activity was measured 48 h after the infection. Similar to the effect of leptomycin B (LMB), the inhibition of HIV-infection by RaTA was dose-dependent from concentrations of 0.1 nM up to 10 to 100 nM above which no luciferase signal could be detected (Figure 2A and 2B). Cell toxicity appeared at concentrations above 1 nM. Normalized mean luciferase values (% of DMSO control) from two independent experiments were used to evaluate relative drug potency. The calculated EC_50_ and CC_50_ for RaTA were around 1.7 nM and 4.6 nM respectively, and 6.8 nM and 16 nM for LMB. Although the calculated SI value was similar for both drugs (2.7 for RaTA and 2.3 for LMB), RaTA exhibited an overall 4-fold higher potency than LMB in the TZM-bl cells assay (Figure 2A and 2B).

It is known that luciferase-mRNA requires the CRM1-nuclear export pathway to be expressed [29]. Since the TZM-bl infection-assay is based on the luciferase expression induced upon HIV infection [28], the calculated inhibitory values for the test compounds could be biased. To confirm that the observed antiviral activity of RaTA was not due to an unspecific luciferase inhibition, we tested the effect of RaTA on the production of the HIV capsid protein p24 in a Western Blot (Figure 2C and 2D). At 10 nM RaTA, the HIV p24 production was reduced to about 10% relative to the solvent control. This inhibition is identical to that measured in the TZM-bl assay (Figure 2A), thus excluding an unspecific artefact from the assay system used.

### Ratjadone A decreases HIV p24 production in MT-2 cells

**Figure 3:**
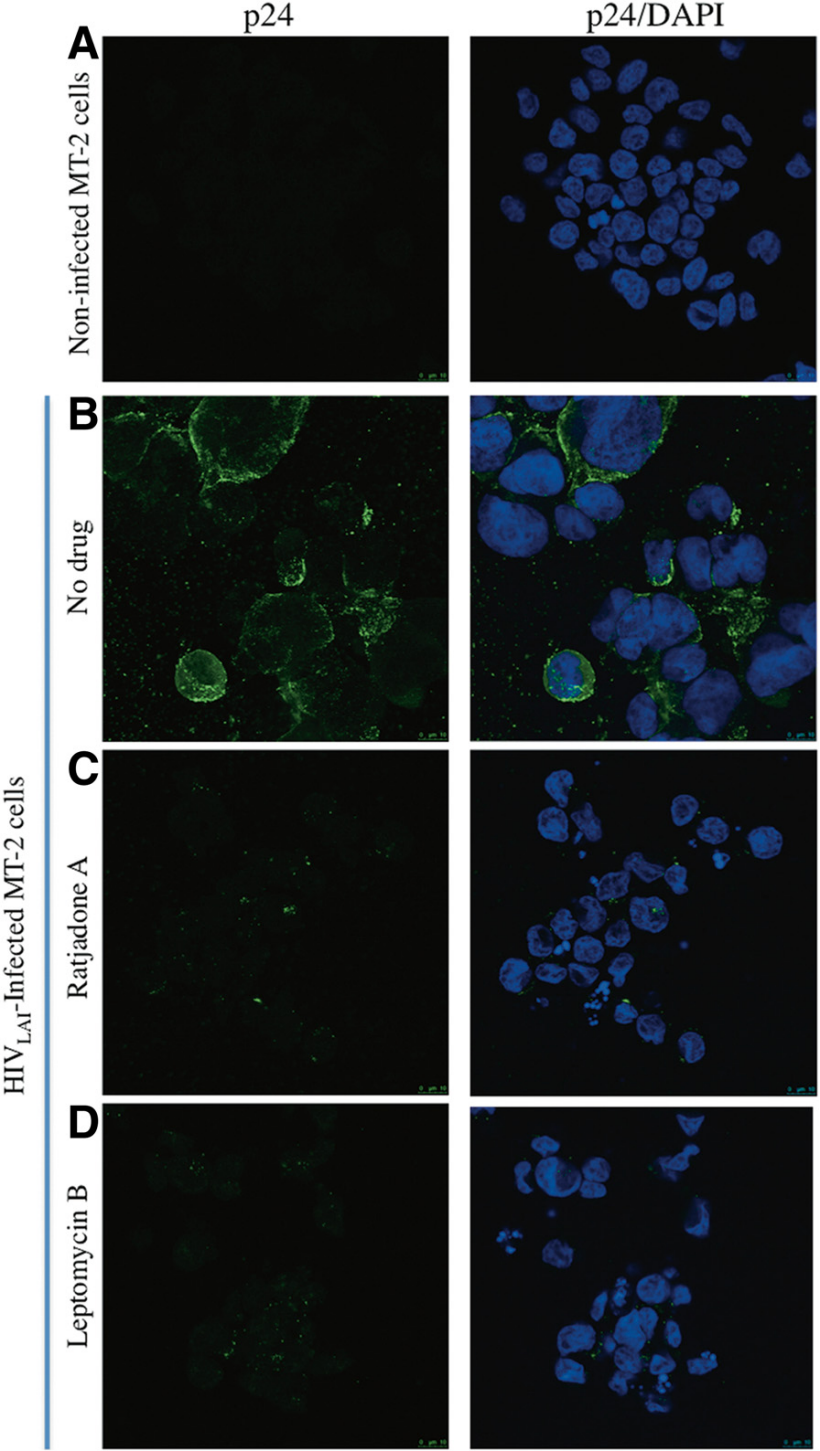
**Ratjadone A inhibits HIV p24 expression in MT-2-infected cells.** MT-2 cells were infected with HIV_LAI_ and treated with ratjadone A or leptomycin B. 48 h after infection cells were fixed and stained for HIV-p24 protein (green signals) and with DAPI (blue signals). Not infected and infected but untreated cells were used as negative and positive controls respectively. **(A)** non-infected cells. **(B)** p24 distribution in infected cells without drugs. **(C)** p24 distribution in infected cells treated with ratjadone A, and **(D)** p24 distribution in infected cells treated with leptomycin B.

To further confirm the antiviral activity of RaTA in a T-cell line, MT-2 cells were pre-treated with RaTA or LMB at concentrations of 2 nM and 4 nM respectively or left untreated (MOCK control), and infected with HIV. After 48 h, HIV p24 production was analysed by immunofluorescence. Un-infected control cells showed very low background signals (Figure 3A), compared to the intense p24 signals of the infected control cells (Figure 3B). In contrast, treatment with RaTA or LMB significantly inhibited HIV p24 production (Figure 3C-D). Although it has been described that drug EC_50_ estimations vary among different cell types, these results are consistent with the HIV inhibition observed in the TZM-bl cell assay and further ruled out the possibility of unspecific effects on luciferase expression and activity.

### Ratjadone A inhibits HIV at the mRNA nuclear export step

**Figure 4:**
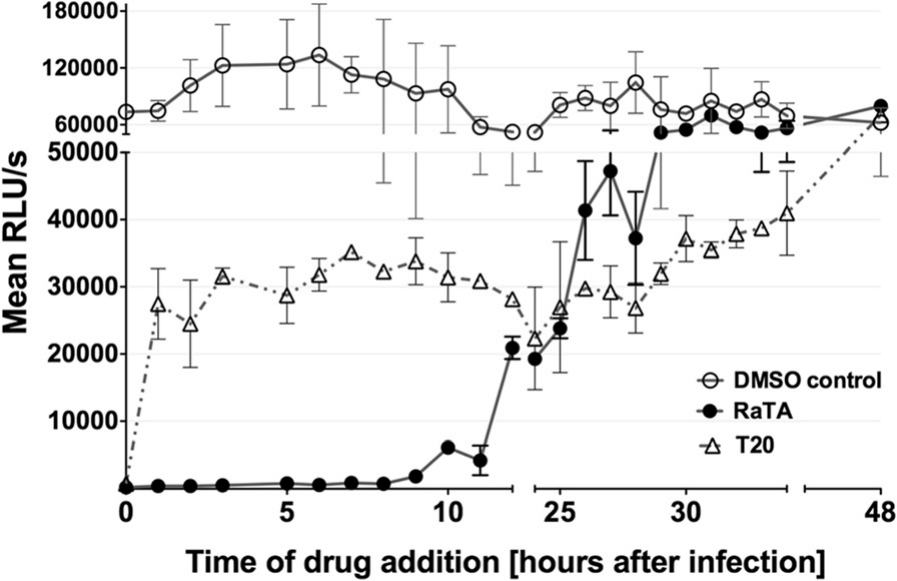
**Ratjadone A blocks HIV infection at a time point corresponding to viral mRNA nuclear export.** TZM-bl cells were synchronously infected with HIV_LAI_ and plated in 96-wells plates in duplicate. Then 10 nM ratjadone A or 1 μM T20 were added at the indicated time points. The drug solvent (0.1% DMSO) was used as control. 72 h after infection luciferase activity was measured for every time point. Values are plotted as relative light units per seconds of exposure and are the mean of the duplicates. Error bars are standard error of the mean (SEM).

To corroborate that the step of the replication cycle targeted by RaTA is the nuclear export of HIV mRNAs, we performed a time-of-drug-addition experiment (TOA) in TZM-bl cells. TOA is a critical assay that helps identify the mode of action and putative secondary targets of new antiviral drugs [30]. In this assay, drugs are added at different time points after synchronized infections and the drug’s inhibitory effect on viral replication is measured 72 h later. A drug-mediated viral inhibition is visible up to a time point corresponding to the replication step targeted by the drug. If the drug is added after it’s targeted replication step, viral replication will not be inhibited and a “jump” in the resulting infectivity curve will be seen [30,31]. Briefly, cells were spinoculated with HIV at 4°C, washed and dispensed into 96-well plates. Infection was synchronized by addition of culture medium at 37°C and drugs were added at the indicated time points. The infectivity curves are shown in Figure 4. The anti-HIV action of RaTA occurs around 12 h after synchronized infection, a time-point coinciding with the HIV-RNA nuclear export step [31]. The HIV-entry inhibitor Enfuvirtide T20 was used as a positive control for the TOA assay. T20 has a well-defined mechanism of action that results in a characteristic TOA infectivity curve [31]. Our results were comparable (Figure 4) demonstrating the accuracy of our experimental conditions and assay outputs. Variations seen in the infection recovery after the time of drug action are commonly observed in TOA experiments and can be due to residual effects of the drugs when targeting a repetitive process that takes place for hours [31]. Depending on factors such as affinity and concentration, individual drugs can have more than one effective target, thus affecting additional steps in the virus replication cycle. It has been described that increasing concentrations of an antiviral drug might saturate its primary target and that residual unbound drug can then be available for blocking additional viral processes [31]. This effect will be evident in TOA by a change in the shape of the time-response curve. At the concentration tested here, RaTA does not seem to target additional steps of the HIV replication cycle.

### Ratjadone A inhibits the Rev-dependent HIV mRNA nuclear export

**Figure 5:**
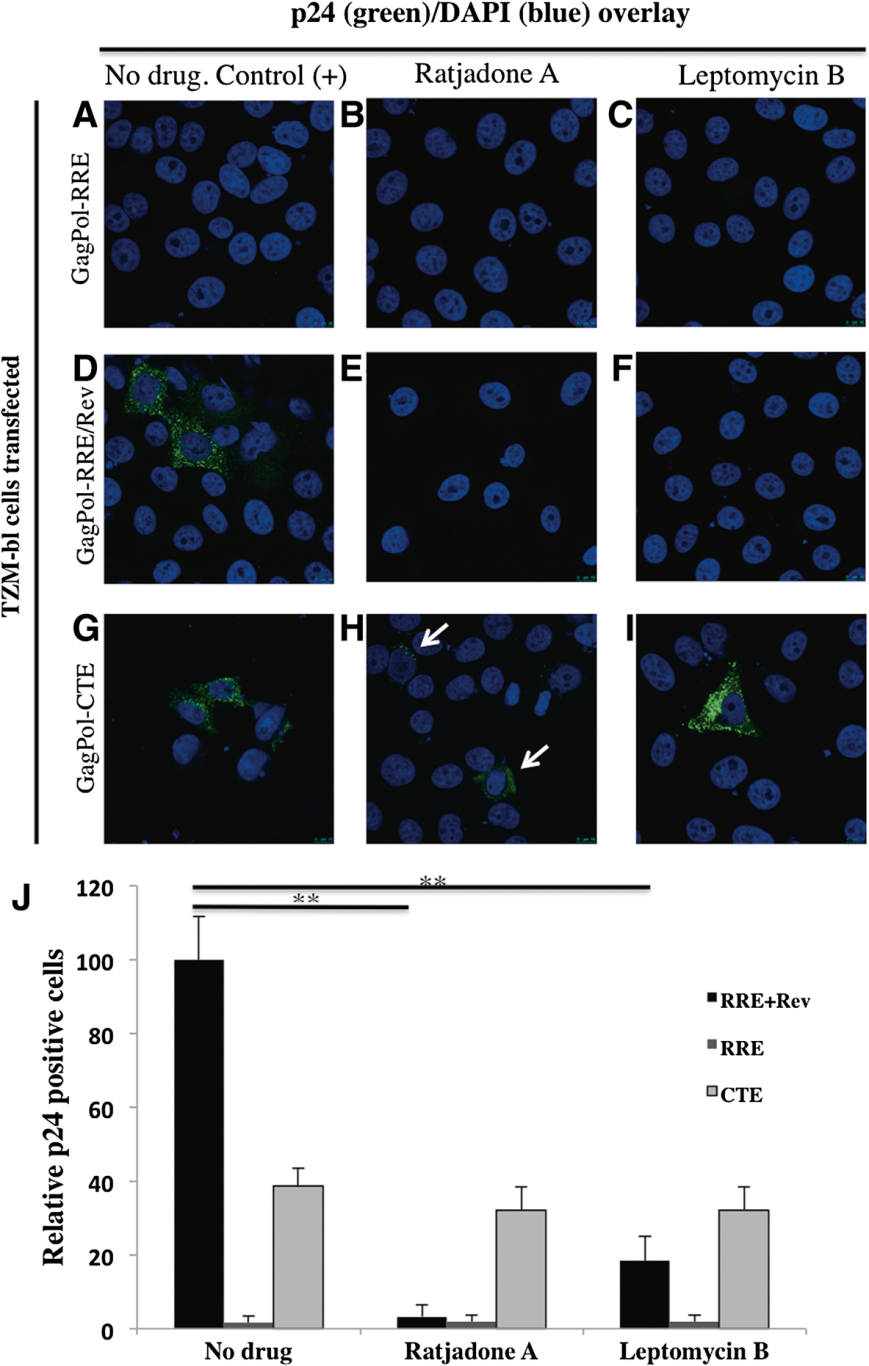
**Ratjadone A inhibits the Rev-dependent HIV-RNA nuclear export.** TZM-bl cells were transfected with a GagPol-RRE plasmid **(A to C)**; co-transfected with GagPol-RRE and Rev expression plasmids **(D to F)** or with a GagPol-CTE expression plasmid **(G to I)**. Cells were left untreated or incubated with the indicated drugs at 2 nM (RaTA) and 4 nM (LMB) concentrations. 48 h after transfection cells were fixed and stained for HIV-p24 protein (green signal) and DAPI (blue signal). Samples **(A)** to **(C)** serve as negative controls, and **(D)** as positive control. Arrows in **(H)** indicate p24 positive cells. **(J)** The percentage of p24 positive cells for every sample is shown. Significant differences with p < 0.01 are marked with asterisks. Error bars are standard error of the mean (SEM) of p24-positive cells counted in 10 microscopic fields.

To test if ratjadone A specifically blocks the Rev-dependent HIV mRNA nuclear export, TZM-bl cells transfected with plasmids pCMVGagPol-RRE (Rev-dependent nuclear export), pCMVGagPol-CTE (Rev-independent nuclear export) and pCMVRev were incubated with RaTA or LMB (as a control) and assayed for p24 expression by immunofluorescence (Figure 5). As expected, cells transfected with pCMVGagPol-RRE alone did not show p24 signals (Figure 5A-C). Levels of p24 from mock-treated cells co-transfected with pCMVGagPol-RRE and pCMVRev plasmids (Figure 5D) were set to 100% and relative inhibitory values were calculated (Figure 5J). RRE-Rev transfected cells treated with RaTA (Figure 5E) or LMB (Figure 5F) exhibited a significant decrease (to around 3% and 20% of the untreated control, respectively) in p24 expression (p < 0.01 in both cases) (Figure 5J). The observed lower levels of p24 expression by the pCMVGagPol-CTE plasmid (around 30-40% of the control; Figure 5J) have been noted in previous studies [32]. Despite this, p24-positive cells were visible with or without drug addition (Figure 5G-I). These results show that RaTA inhibition is specific for the Rev-mediated HIV mRNA nuclear export pathway.

### Ratjadone A interacts with CRM1 but not with Rev

**Figure 6:**
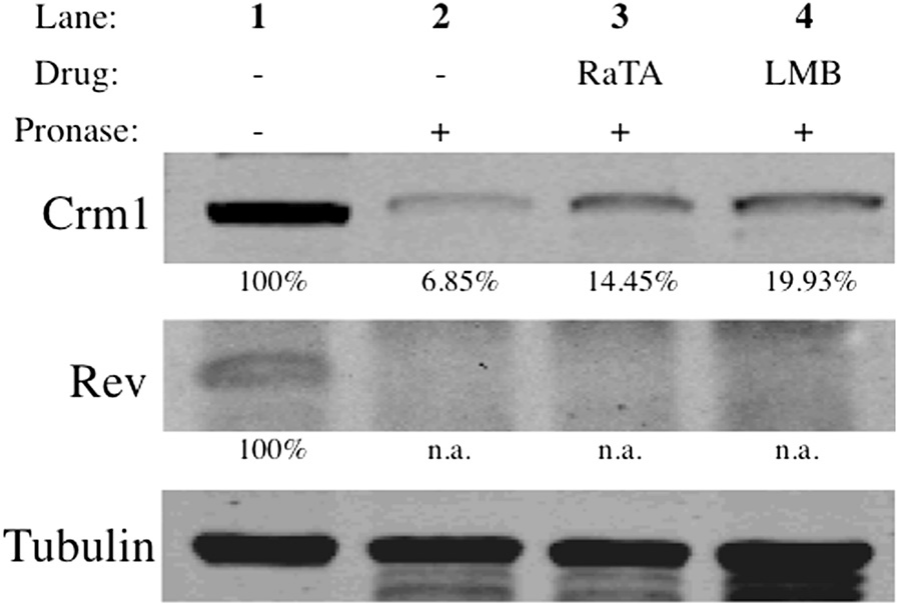
**Ratjadone A protects CRM1 from proteolysis.** Lysates from Rev-transfected 293 T cells were mock-treated with DMSO or incubated with 200 nM ratjadone A or leptomycin B and digested with Pronase. Samples were loaded on a SDS-PAGE gel and developed by Western Blot (see Materials and methods). Lane 1: undigested DMSO control; Lane 2: digested DMSO control; Lane 3: digested sample incubated with RaTA; Lane 4: digested sample incubated with LMB. Percentages of protection relative to undigested controls are shown below the protein bands. Both RaTA and LMB protect CRM1 (upper row) but not Rev (middle row) from degradation. Tubulin was used as a loading control (lower row). The drug solvent concentration (0.1% DMSO) in every sample was constant. Abbreviations: RAT = Ratjadone A; LMB = Leptomycin B; DMSO = dimethyl sulfoxide; n.a. = not applicable.

In order to investigate binding of RaTA to CRM1 and/or Rev, we performed a drug affinity responsive target stability (DARTS) assay. DARTS is based on the principle that a protein is in a dynamic equilibrium with alternative conformations. Ligand binding, mediated by hydrophobic, hydrogen bonding and/or electrostatic interactions, then favors a specific conformation so that upon saturation with a specific ligand, the equilibrium shifts towards the ligand bound conformation. This leads to a thermodynamically more stable state in which resistance to protease degradation is markedly increased [33,34]. To test this in the context of CRM1-Rev interaction, lysates from Rev-transfected 293 T cells or HIV-infected MT-2 cells were incubated with RaTA, LMB (positive control) or DMSO (vehicle control). Samples were digested with a cocktail of proteases and subjected to Western Blotting using specific anti-CRM1 and anti-Rev antibodies. The Western Blot bands of the target protein incubated with the drug should be more intense than those of the target protein incubated with DMSO as control. As shown in Figure 6, both RaTA and LMB exert a clear protection band of around 110 kDa that corresponds to CRM1 (Figure 6, lanes 3 and 4). The relative protection of CRM1 exerted by the drugs was more than 2-fold higher compared to the DMSO control (Figure 6, lane 2). However, there was no significant protection of Rev (around 19 kDa) by the test compounds. Rev appears as 2 close distinct bands in the positive control (Figure 6, lane 1). This 1-2 kDa shift could be due to the partial phosphorylation of Rev as described before [35-37]. No differences were observed when comparing RaTA- or LMB-treated samples with the untreated control (Figure 6, lane 2, 3 and 4 of Rev). At the pronase to protein ratio used in our assay (1:1000), tubulin is not significantly degraded. Therefore, it can be used as loading control [33]. Similar results were obtained using lysates of HIV-infected MT-2 cells (data not shown). These observations indicate that both RaTA and LMB exert their anti-HIV activity by binding to CRM1 but not to Rev.

## Discussion

The Rev-mediated HIV nuclear export step is an attractive target for the development of new antivirals. Besides its regulatory function, Rev has been associated with pathogenesis as Rev-independent SIV clones seem to be attenuated in monkeys [38]. Some argue that even a partial blockade of Rev may contribute to virus control *in vivo*[8]. So far, compounds directly acting on Rev-RRE have low rates of viral inhibition [12,39] and are likely to generate drug-resistance mutants [40,41]. In a recent study, Naji et al. found a strikingly high amount of cellular proteins interacting with Rev that could also be explored as targets for putative new anti-retroviral strategies [42]. The targeting of host-factors is an appealing alternative to conventional antiviral drugs [43-49]. Compounds blocking viral replication by acting on cellular components might reduce the likelihood of resistance development and, if promiscuously used by several viruses, may even exert broad-spectrum antiviral properties.

HIV uses the CRM1-mediated pathway for export of RRE-containing viral mRNAs and the viral genomic RNA out of the nucleus [2]. Remarkably, the CRM1-dependent nuclear export pathway is shared by HIV and HTLV [9,50-52], and it has been suggested to be preferentially used by other clinically relevant viruses such as Dengue Virus [53] and Hepatitis C virus [54]. Although these interesting findings mark CRM1 as a putative good target for broad-spectrum antivirals, the associated toxic effects of CRM1 inhibitors have discouraged their further research. The myxobacterial polyketide ratjadone A blocks the CRM1-mediated nuclear export of mRNAs with a similar mode of action as the related inhibitor leptomycin B [26,27,55]. Although the basis of the CRM1-inhibition by ratjadones is known, their effectiveness against HIV infection was not previously defined. Here we show that ratjadone A inhibits HIV at the nanomolar range with a higher intrinsic potency than leptomycin B. Ratjadone A blocks the CRM1/Rev-mediated mRNA nuclear export by binding to CRM1 but not to Rev. These results suggest that treatment with ratjadones might not induce resistance development, although further studies are needed to corroborate this hypothesis.

Recently, it has been shown that sensitizing cells by blocking CRM1 with low concentrations of ratjadone improves the response to topoisomerase II treatment in multiple myeloma cancer cells [56], and that related CRM1 inhibitors are effective in T-cell leukaemic *in vivo* models [57]. Likewise, CRM1 inhibitors may be tested as a component in alternative combination therapies against viral diseases. For this, the study of ratjadone derivatives that exhibit diverse efficacies of blocking CRM1 [27] could be an interesting direction to follow.

## Conclusions

The myxobacterial metabolite ratjadone A is an efficient HIV inhibitor by blocking the nuclear export protein CRM1. Due to its mode of CRM1 inhibition and the low selectivity index, the potential use of ratjadone A as a mono-therapeutic antiviral is very limited. However, since host-acting drugs are unlikely to generate resistance, further studies including derivatives of ratjadones in combination with ART might help devise alternative antiretroviral therapies in the future.

## Materials and methods

### Cell culture

293 T cells (ATCC, CRL-11268) were maintained at 37°C and 5% CO_2_ in Dulbecco’s modified Eagle’s medium (DMEM) (Gibco, Paisley, UK) supplemented with 10% heat-inactivated fetal calf serum (FCS) and 1% penicillin-streptomycin. TZM-bl cells (NIH AIDS Research and Reference Reagent Program, catalogue number: 8129) were maintained with DMEM supplemented with 10% heat-inactivated FCS, HEPES 25 mM and 0.5% Gentamycin. MT-2 (NIH AIDS Research and Reference Reagent Program, catalogue number: 237) and PM1 cells (NIH AIDS Research and Reference Reagent Program, catalogue number: 3038) were maintained with RPMI medium supplemented with 10% heat-inactivated FCS and 1% of penicillin-streptomycin.

### Plasmids

Plasmids pCMVGagPol-RRE, pCMVGagPol-CTE (a kind gift from Kuan-Teh Jeang, NIAID, NIH, USA [58]) and pCMV-Rev were used. Plasmid pCMVGalPol-RRE contains the HIV-GagPol region and the Rev-responsive element (RRE), pCMVGagPol-CTE contains the HIV-GagPol RNA region and the Constitutive Transport Element (CTE) from the Mason-Pfizer Monkey Virus (MPMV), and pCMV-Rev expresses the HIV-Rev protein. Transfections in HEK 293 T and TZM-bl cells were performed with Lipofectamine 2000 (Invitrogen, Paisley, UK) according to the manufacturer’s manual. A pSV2-gpt expression plasmid was used as negative control.

### Drugs

Ratjadone A is from the collection of myxobacterial secondary metabolites of the Helmholtz Centre for Infection Research, Braunschweig, Germany. Leptomycin B was purchased from Sigma, St. Louis, USA. The entry inhibitor Enfuvirtide (Fuzeon, Roche, Basel Switzerland) was used as a positive control for the time-of-drug addition experiments (TOA).

### Virus stocks

HIV-1_LAI_ isolate was obtained from the Centre for AIDS Reagents, NIBSC (UK). Virus was propagated in PM1 cells, titrated and stored at -80°C. Infections were performed in triplicate at a multiplicity of infection (MOI) of 0.5. Infected TZM-bl cells were used to obtain EC_50_ and CC_50_ values and for TOA (see below) experiments. MT-2 cells were infected for immunostaining and DARTS assay (Section 2.8).

### Dose-response assays

TZM-bl cells were plated (10^4^ cells/well) in Nunc® MicroWell 96 well optical bottom plates (Sigma) and incubated for 1 h with increasing concentrations of test compounds in 10-fold dilutions or with the corresponding vehicle (DMSO or MeOH) as negative control in triplicates. After drug incubation, cells were infected with HIV_LAI_ at MOI = 0.5. 48 h after infection luciferase activity was measured using Britelite Plus™ (PerkinElmer, Waltham, USA). In parallel, cell viability of TZM-bl cells was determined with an ATP quantification method using the commercial kit CellTiter-Glo® Luminescent Cell Viability Assay (Promega, Madison, USA). ATP is a marker of the presence of metabolically active cells [59]. Therefore, the ATP levels relative to the untreated control are a measure of drug-induced cytotoxicity. Mean luciferase values were normalized to untreated controls and Effective Concentration 50 (EC_50_) and Cytotoxic Concentration 50 (CC_50_) were calculated in GraphPad Prism (GraphPad Software, San Diego, CA, USA) by analyzing the log_dose_ vs. normalized response. The Selectivity Index (SI) refers to the antiviral potency of a drug and is calculated as the ratio of CC_50_ to EC_50_[60,61].

For western blotting, proteins of cell lysates were separated on a SDS-PAGE and transferred to nitrocellulose membranes (Whatman, Dassel, Germany). Western Blots were developed with anti-HIV-1 p24 (NIBSC, UK) and anti-mouse IgG-Horseradish peroxidase (GE Healthcare, Bio-Sciences, Little Chalfont, UK) as primary and secondary antibodies, respectively. Bands were detected using SuperSignal® West Femto Maximum Sensitivity Substrate (Thermo scientific, Waltham, USA) and visualized with a LumiImager (LAS-1000). Protein bands were quantified by densitometric scanning using Image Gauge software (Fuji Photo Film Co, Ltd, Tokyo, Japan).

### Immunofluorescence

TZM-bl cells were plated on poly-L-lysine-coated microscopy glasses (Thermoscientific) and transfected with plasmids pCMVRev, pCMVGagPol-RRE or pCMVGagPol-CTE. 48 h after transfection, glasses were fixed in PBS containing 4% paraformaldehyde. After permeabilizing (20 minutes in 0.2% Triton X-100; Sigma) and blocking (30 minutes with FCS 10%), samples were stained for 1 h with anti-HIV p24 antibody provided by the Centre for AIDS Reagents, NIBSC (UK), and a secondary anti-mouse IgG antibody labelled with Alexa Fluor 647 (Invitrogen) for 45 minutes in the dark, followed by a 15 minutes nuclear staining with DAPI. Glasses were placed in microscopy slides with Mowiol (Sigma) and examined with the Leica TCS SP2 confocal microscope at 40× magnification (to count cells) and Leica TCS SP5 at 63× (to take pictures). MT-2 cells were infected and stained following the same protocol.

### Time of drug addition experiment (TOA)

The TOA protocol was performed as described [30,31]. Briefly, TZM-bl cells (10^6^ cells) were synchronously infected with HIV_LAI_ virus at a MOI of 0.5 by spinoculation at 4°C for 90 minutes at 300 g [62]. After three washing steps with cold PBS to remove unattached virus, cells were plated into a 96-well optical plate and the infection was synchronized through adding culture medium at 37°C, defining time 0 h of the experiment. Then 10 nM of ratjadone A was added at the indicated time points post-infection. 1 μM of T20 was added as a control. Luciferase activity was measured with Britelite Plus™ (PerkinElmer) 72 h after infection.

### Drug affinity responsive target stability (DARTS) assay

The DARTS assay was developed by Lomenick et al. [33] and is based on the property of small molecules to protect their targets from protease degradation. Briefly, Rev-transfected 293 T cells and HIV-infected MT-2 cells were lysed with M-Per lysis buffer (Thermoscientific) containing a mixture of protease inhibitors to avoid endogenous protein degradation. Lysates were incubated at room temperature for 1 h either with RaTA, LMB or left untreated, and digested with Pronase (Roche) at a protease to protein ratio of 1:1000 for 30 minutes. Digestion was stopped by addition of SDS-PAGE loading buffer (with SDS and mercaptoethanol) and heating for 5 minutes at 95°C [33]. Samples were run on a SDS-PAGE electrophoresis gel and were transferred to nitrocellulose membranes (Whatman, Dassel, Germany). Western Blots were developed with anti-CRM1 (Abcam, Cambridge, UK) and anti-α-tubulin (SIGMA) as primary antibodies. Anti-rabbit or anti-mouse fluorescent-labelled antibodies (LI-COR, Lincoln, USA) were used as secondary antibodies. Membranes were visualized with a scanner Odyssey®CLx and analyzed by densitometric scanning of the bands with the Odyssey software V3.0 (LI-COR). Rev bands were stained with anti-Rev (NIBSC, UK) and anti-mouse IgG-Horseradish peroxidase (GE Healthcare, Bio-Sciences, Little Chalfont, UK) as primary and secondary antibody, respectively. Bands were detected using SuperSignal® West Femto Maximum Sensitivity Substrate (Thermo scientific, Waltham, USA) and visualized as described above.

## Abbreviations

CRM1: Chromosome Region Maintenance 1.

## Competing interests

The authors declare that they have no competing interests.

## Authors’ contributions

EFS, JPM and BH performed the HIV inhibition experiments and analysed the data; KG grew myxobacteria for ratjadone production; PW purified ratjadone; JPM, JD, RF, FS and AM designed experimental strategy and wrote the manuscript. All authors read and approved the final manuscript.
